# Considerations for a More Ethical Approach to Data in AI: On Data Representation and Infrastructure

**DOI:** 10.3389/fdata.2020.00025

**Published:** 2020-09-02

**Authors:** Alice Baird, Björn Schuller

**Affiliations:** ^1^Chair of Embedded Intelligence for Health Care and Wellbeing, University of Augsburg, Augsburg, Germany; ^2^Group on Language, Audio & Music, Imperial College London, London, United Kingdom

**Keywords:** artificial intelligence, machine learning, ethical AI, decentralization, selection-bias

## Abstract

Data shapes the development of Artificial Intelligence (AI) as we currently know it, and for many years centralized networking infrastructures have dominated both the sourcing and subsequent use of such data. Research suggests that centralized approaches result in poor representation, and as AI is now integrated more in daily life, there is a need for efforts to improve on this. The AI research community has begun to explore managing data infrastructures more democratically, finding that decentralized networking allows for more transparency which can alleviate core ethical concerns, such as selection-bias. With this in mind, herein, we present a mini-survey framed around data representation and data infrastructures in AI. We outline four key considerations (*auditing, benchmarking, confidence and trust, explainability and interpretability*) as they pertain to data-driven AI, and propose that reflection of them, along with improved interdisciplinary discussion may aid the mitigation of data-based AI ethical concerns, and ultimately improve individual wellbeing when interacting with AI.

## 1. Introduction

Artificial intelligence (AI) in its current form relies heavily on large quantities of data (Yavuz, 2019), and data-driven Deep Neural Networks (DNNs) have prompted fast-paced development of AI (Greene, 2020). Currently, the research community is under great strain to keep up with the potential ethical concerns which arise as a result of this (Naughton, 2019). Within the AI community such ethical concerns can require quite some disentanglement (Allen et al., 2006), and it is not until recently that AI-based research groups have begun to provide public manifestos concerning the ethics of AI, e.g., Google's DeepMind, and the Partnership AI.^1^

The *Ethics of AI* (Boddington, 2017) is now an essential topic for researchers, both internal and external, to core-machine learning and differs from *Machine Ethics* (Baum et al., 2018). The latter refers to giving conscious ethical based decision-making power to machines. The *Ethics of AI*, although somewhat informing *Machine Ethics*, refers more broadly to decisions made by researchers and covers issues of diversity and representation, e.g., to avoid discrimination (Zliobaite, 2015) or inherent latent biases (van Otterlo, 2018). Herein, our discussion focuses on topics relating to the *Ethics of AI* unless otherwise stated.

There has been recent research which shows promise for improved data learning from smaller quantities (“merely a few minutes”) of data (Chen et al., 2018). However, machine learning algorithms developed for AI commonly require substantial quantities of data (Schneider, 2020). In this regard, *Big Data* ethics for AI algorithms are an expanding discussion point (Berendt et al., 2015; Mittelstadt and Floridi, 2016). Crowdsourcing (i.e., data gathered from large amounts of paid or unpaid individuals via the internet), is one approach to collect such quantities of data. However, ethical concerns including worker exploitation (Schlagwein et al., 2019), may have implications on the validity of the data. Additionally researchers utilize *in-the-wild* internet sources, e.g., YouTube (Abu-El-Haija et al., 2016) or Twitter (Beach, 2019), and apply unsupervised labeling methods (Jan, 2020). However, in Parikh et al. (2019), the authors describe how approaches for automated collection and labeling can result in the propagation of historical and social biases (Osoba and Welser IV, 2017). In the health domain, such bias could have serious consequences, leading to misdiagnosis or incorrect treatment plans (Mehrabi et al., 2019).

One method to avoid bias in AI is through the acquisition of diverse data sources (Demchenko et al., 2013). With *Veracity* (i.e., habitual truthfulness) being one of the *5 Vs* (e.g., Velocity, Volume, Value, Variety and Veracity) for defining truly Big Data (Khan et al., 2019). However, big data is commonly, stored in *centralized* infrastructures which limit transparency, and democratic, decentralized (i.e., peer-to-peer blockchain-based) approaches are becoming prevalent (Luo et al., 2019).

Centralized data storage can be efficient and beneficial to the “central” body to which the infrastructure belongs. However, it is precisely this factor amongst others (i.e., proprietary modeling of underrepresented data) that are problematic (Ferrer et al., 2019).

Furthermore, centralized platforms limit the access and knowledge that data providers receive. The General Data Protection Regulation (GDPR) was established within the European Union (The-European-Commission, 2019) to partly tackle this. GDPR is a set of regulations of which the core goal is to protect the data of individuals that are utilized by third parties. In its current form, GDPR promotes a centralized approach, supporting what are known as *commercial governance platforms*. These platforms control restrictions to employees based on a data providers request but primarily function as a centralized repository. In essence, GDPR meant that companies needed to re-ask for data-consent more transparently. However, the “terms of agreement” certificate remains the basis, and 90 % of users are known to ignore its detail (Deloitte, 2016).

**Figure 1:**
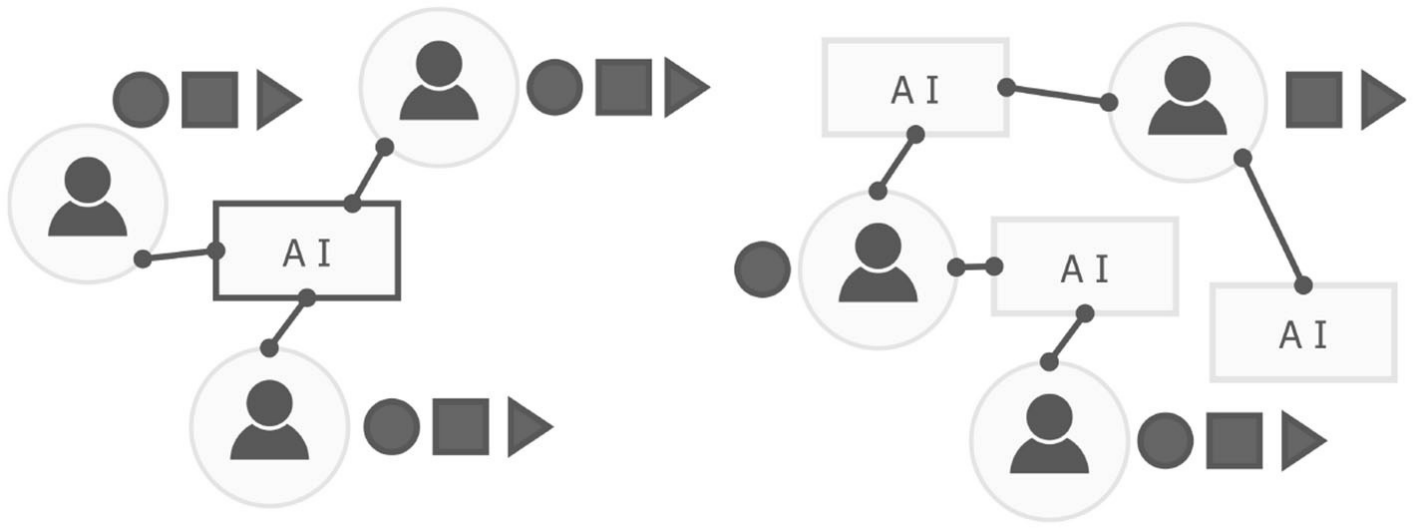
A simplified overview of a typical centralized (left) and decentralized (right) network infrastructure. In the right figure individuals choose the modality to share (as indicated by circle, square, and triangle icons), and users in the network have agency in how their data is used. In the left figure, the AI is essentially a black-box, and users make all modalities of data available to all components of the AI infrastructure.

As a counter approach to the centralized storage of data, for some time researchers have proposed the need for a *decentralized* (cf. Figure 1) networking in which individual data is more easily protected (i.e., there is no “single point” of failure). In this infrastructure, individuals have more agency concerning the use of their data (Kahani and Beadle, 1997). Primarily, individuals choose to access parts of a network rather than its entirety. On a large scale, this paradigm would remove the known biases of centralized networks, as targeted collection, for example, would be less accessible by companies and sources of the data more complex to identify. In this way, various encryption algorithms, including homomorphic encryption (a method which allows for data processing while encrypted), or data masking, are being integrated within decentralized networks, allowing for identity preservation (Setia et al., 2019). Federated Learning (FL) (Hu et al., 2019), is one approach which can be applied to decentralized networks to improve privacy (Marnau, 2019). In FL, weights are passed from the host device and updated locally, instead of raw data leaving a device (Yang et al., 2019).

With these topics in mind, in this contribution, we aim to outline core ethical considerations, which relate to data and the ethics of AI. Our focus remains on the ethics of data representation and data infrastructure, particularly *selection-bias* and *decentralization*. We chose these topics due to their common pairing in the literature. A regular talking-point in machine learning is *selection-bias* and a networking infrastructure which may help to more transparently observe this is *decentralization* (Swan, 2015; Montes and Goertzel, 2019).

Our contribution is structured as follows; firstly we shortly define key terminology used throughout the manuscript in section 2, followed by a brief background and overview of the core themes as they pertain to AI in section 3. We then introduce our ethical data considerations in section 4 providing specific definitions and general ethical concerns. Following this in section 5, we connect these ethical considerations more closely with data representation and infrastructure, and in turn, outline technical approaches which help reduce the aforementioned ethical concerns. Finally, we offer concluding remarks in section 7.

## 2. Terminology

There are a variety of core terms which are used throughout this manuscript which may have a dual meaning in the machine learning community. For this reason, we first define here three core terms, *ethics, bias*, and *decentralization* used within our discussion.

As mentioned previously, we focus on the *Ethics of AI* rather than *Machine Ethics*. However, further to this, we use the term *ethics* based on guidelines within applied ethics, particularly in relation to machine understanding. In Döring et al. (2011), the principles of *beneficence, non-maleficence, autonomy*, and *justice* are set out as being fundamental considerations for those working in AI. Although this is particular to emotionally aware systems, we consider that such principles are relevant across AI research. Of particular relevance to this contribution, is autonomy, i.e., a duty for systems to avoid interference, and respect an individual's capacity for decision-making. This principle impacts upon both *data representation* and *infrastructure* choices (e.g., centralized or decentralized).

We consistently refer to the term *bias* throughout our contribution. First introduced to machine learning by Mitchell (1980), we typically discuss statistical biases, unless otherwise stated, which may include absolute or relative biases. To be more specific, we focus closely on data in this contribution, and therefore dominantly refer to *selection-bias*. *Selection-bias* stems in part from prejudice-based biases (Stark, 2015). However, *selection-bias* falls within statistical biases as it is a consequence of conscious (hence prejudice) or unconscious data selection. *Selection-bias* is particularly relevant to AI given that real randomization (or diverse representation) of data is not always possible.

As a critical aspect of our contribution, relating to the mitigation of bias, through a more ethical approach to data infrustructure, we consistently refer to *decentralized* AI. A broad definition of *decentralization* is the distribution of power moving away from central authorities. In the context of AI, when discussing *decentralization*, we refer to decentralized architectures which allow for this type of distribution, in regards to data sourcing, management and analysis. We do touch on literature relating to blockchain, which is a well-known decentralized approach. However, the term is utilized here more generally and is not exclusive to the blockchain.

## 3. Background: Bias and Decentralization in AI

Funding and global research efforts in the field of AI have increased in the last decade, particularly in the areas of health, transportation, and communication (Mou, 2019). Along with this increase has come a rise in ethical demands related to Big Data (Herschel and Miori, 2017). Although *true* Big Data is said to need *Veracity*, the reality of this is sometimes different, with large-scale data often showing particular biases toward clustered demographics (Price and Ball, 2014). As a result, terms, such as *Machine Learning Fairness*—promoted initially by Google Inc.^2^—is now regularly referred to in an endeavor to build *trust* and show ethical sensitivity (Mehrabi et al., 2019). In this regard, IBM released their AI Explainability 360 Toolkit^3^ in which the overarching goal appears to be improving *trust* in AI, through more deeply researching machine learning biases, as it pertains to the research areas of fairness, robustness and *explainability*.

Three common forms of bias are discussed concerning AI, i.e., interaction-bias, latent-bias, and *selection-bias*. *Selection-bias* occurs when the data used within a paradigm is selected with bias, leading to misrepresentation rather than generalization. In particular, researchers are repeatedly finding bias in regards to gender (Gao and Ai, 2009). Wang et al. (2019a) found for example that models tend to have a bias toward a particular gender even when a dataset is balanced—which could point to lower level architecture-based biases (Koene, 2017). *Selection-bias* is essential to combat when referring to models developed for human interaction. Based on data decision making, a bias can propagate through system architectures, leading to lower accuracy on a generalized population. Lack of generalization is particularly problematic for domains, such as health, where this may result in a breach of patient safety (Challen et al., 2019).

Furthermore, the evaluation of *fairness* in machine learning is another prominent topic, highlighted as a machine learning consideration in Hutchinson and Mitchell (2019). Additionally, researchers propose *fairness metrics* for evaluating the bias which is inherent to a model (Friedler et al., 2019), including the Disparate Impact or Demographic Parity Constraint (DPC). DPC groups underprivileged classes and compares them to privileged classes as a single group. Similarly, there are novel architectures which mitigate bias through prioritization of minority samples, and the authors of this approach suggest that there is an improvement in *generalized fairness* (Lohia et al., 2019).

A core contributing factor to bias in AI is the management of data. Current AI networking is based on centralized infrastructure (cf. Figure 1), where individuals present a unified data source to a central server. This centralization approach not only limits privacy but also creates a homogeneous representation, which is less characteristic of the individual interacting (Sueur et al., 2012).

*Decentralization* in AI was initially coined as a term to describe “autonomous agents in a multi-agents world” (Miiller, 1990), and researchers have proposed *decentralization* for large AI architectures e.g., integrating machine learning with a Peer-to-peer style blockchain approach Zheng et al., 2018] to improve *fairness* and *bias* (Barclay et al., 2018). In this architecture, collaborative incentives are offered to the network users and approaches allow for improved identity-representation, as well as more control in regards to data-usage, resulting in more freedom and higher privacy. Furthermore, a decentralized network may inherently be more ethical as more individuals are interacting with and refining the network with agency (Montes and Goertzel, 2019).

For individuals interfacing with AI, privacy is a concern (Montes and Goertzel, 2019). Improving privacy is a core advantage of decentralized data approaches (Daneshgar et al., 2019). In a centralized approach, anonymization processes exist (e.g., that which are enforced by GDPR), although it is unclear how this is consistently applied. To this end, identification of a participant in the data source may not be needed, yet, unique aspects of their character (e.g., how they pronounce a particular word), are still easily identified (Regan and Jesse, 2019).

There are multiple organizations and corporations which focus on the benefits of *decentralization*, including Effect.AI and SingularityNET^4^ Such organizations promote benefits including “diverse ecosystems” and “knowledge sharing.” The Decentralized AI Alliance^5^ is another organization which integrates AI and blockchain, promoting collaborative problem-solving. In general, the term *decentralization* comes not only from technical network logistic but from philosophical “transhuman” ideologies (Smith, 2019). In regards to the latter, *decentralization* promotes the improvement of human-wellbeing through democratical interfacing with technology Goertzel (2007). This democratic view is one aspect of *decentralization* that aids in the reduction of AI bias (Singh, 2018).

Similarly, there are organizations which focus primarily on the challenge of bias in AI, from many viewpoints including race, gender, age, and disability^6^, most of which implement responsible research and innovation (RRI). When applying RRI to the AI community, the aim is to encourage researchers to anticipate and analyse potential risks of their network, and ensure that the development of AI is socially acceptable, needed, and sustainable (Stahl and Wright, 2018). Biases are an essential aspect of AI RRI (Fussel, 2017), as poor identity-representation has dire consequences for real-world models (Zliobaite, 2015).

## 4. Methodology: Ethical Data Considerations

**Figure 2:**
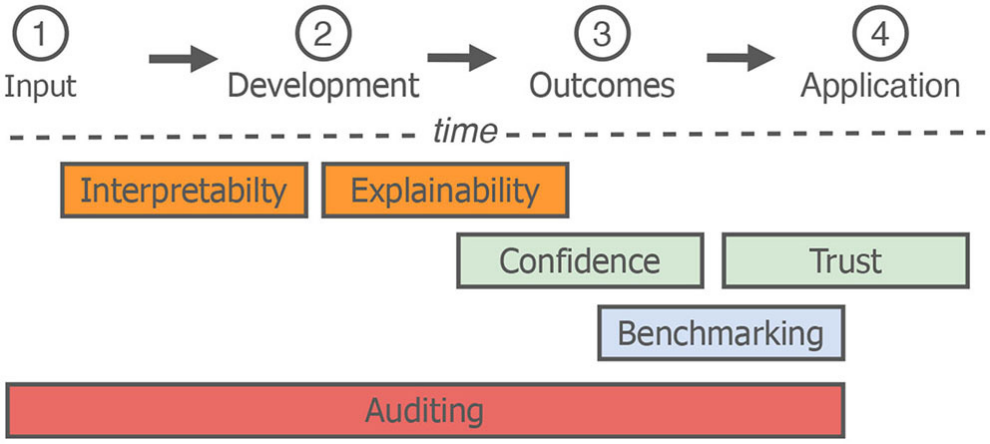
An overview of a machine learning workflow, (1) Data collection and pre-processing, (2) developments of machine learning models, (3) evaluation of model outcomes (i.e., performance), (4) integration of the developed AI in a real-world scenario. We place the four considerations introduced in Section 4 across time. Positions of individual considerations are not static, we define their placement over time, based primarily on their relationship to one another.

There are an array of concerns relating to the ethics of AI, including, joblessness, inequality, security, and prejudices (Hagendorff, 2019). With this in mind, academic and industry-based research groups are providing tools to tackle these ethical concerns (cf. Table 1), mainly based on four key areas. In this section, we introduce and conceptually discuss these four ethical considerations—*auditing, benchmarking, confidence and trust* and *explainability and interpretability*—chosen, due to their prominence within the AI community. As well as this, these four aspects, each have a pivotal impact on data representation, and an inherent relation to data infrastructures. An overview of a typical machine learning workflow with these four considerations highlighted based on their position in time is given in Figure 2. To this end, herein, we first define our four considerations more concretely, followed by a description of specific ethical concerns ([±]) which relate to them.

**Table 1:** Brief overview of prominent ethical AI tools which have been made available by both academic and industry research groups.

**Tool**	**A**	**B**	**E & I**	**C & T**	**Description**
Gender Shades (Buolamwini and Gebru, 2018)	X	X	–	–	An *intersectional* approach to inclusive product testing for AI, relating specifically to gender and race bias.
What-If Tool (Google, 2020)	X	–	X	–	Allows users to analyse their machine learning model through the use of an interactive visual interface.
IBM: AI Explainability 360 Toolkit (Arya et al., 2020)	–	X	X	–	Contains state-of-the-art algorithms that allow for improved interpretability and explainability of machine learning models.
IBM: AI Fairness 360 Open Source Toolkit (Bellamy et al., 2019)	X	–	X	X	Provides a series of metrics for datasets and models to test for biases explicitly, including a clear explanations for those metrics.
LIME (Ribeiro et al., 2016)	–	–	X	X	A general eXplainable-AI toolkit which allows users to reason better for why a model makes certain predictions.
openAI: baseline, Gym, Microscope (Brockman et al., 2016)	–	X	X	–	Provides reproducible reinforcement learning algorithms with benchmarked performances based on published results. As well as visualization methods for observing significant layers and neuron activations.
Procgen: Benchmark (Cobbe et al., 2019)	–	X	–	–	Procedurally-generated environments which provide a benchmark for the speed of a reinforcement learning algorithms generalization.
PwC: Responsible AI Toolkit (Waterhouse Cooper, 2019)	–	–	X	X	A collection of customizable frameworks to harness AI in an ethical and responsible manner.
Pymetrics: Audit AI (Trindel et al., 2019)	X	–	–	–	Contains tools to measure and mitigate the effects of discriminatory patterns, designed specifically for socially sensitive decision processes.

*We highlight their target ethical consideration, namely (A)uditing, (B)enchmarking, (E)xplainability and (I)nterpretabiltiy, (C)onfidence and (T)rust*.

### 4.1. Auditing

In the context of AI data, *auditing* is not dissimilar to research domains, such as economics. An auditor regularly checks aspects of the system, including the data validity itself. For example Fernández and Fernández (2019) propose an AI-based recruiting systems—in which the candidate's data is validated by a manual (i.e., human) auditor. In Figure 2 we have assigned *auditing* to every aspect of the AI workflow, although it is commonly only integrated during earlier development stages.

[±] *Auditing* is integral as acquisition scales up to *Big Data*. The process of managing what Schembera and Durán (2020), describes as “tangible data” can be extremely time-consuming and costly for those involved and human or machine error can propagate, resulting in biases or leading to mostly unusable data (L'heureux et al., 2017). On the other side, is the *auditing* of “dark data.” This data type is estimated to be 90% (Johnson, 2015) of all stored data, and is largely unknown to the user. The literature currently focuses on *auditing* tangible data, as yet there is less attention for dark data (Trajanov et al., 2018).

### 4.2. Benchmarking

In machine learning, *benchmarking* is the process of evaluating novel approaches against well-establish approaches or databases of the same task. To this end, it often comes at a later stage during the AI workflow (cf. Figure 2). In the computer vision domain, this has been particularly successful in pushing forward developments (Westphal et al., 2019), with data sets, such as MNIST (LeCun and Cortes, 2010) or CIFAR-10 (Krizhevsky et al., 2009), continuously benchmarked against in both an academic and industry setting. Pre-trained networks are another *benchmarking* tool. Networks, such as imageNet (Simon et al., 2016) are well-known and consistently applied, given the quantity of data and promising results (Wang et al., 2019d).

[±] Multimodal analysis is becoming more ubiquitous in machine learning (Stappen et al., 2020), due to well-known and longstanding advantages (Johnston et al., 1997). When datasets are multimodal *benchmarking* improvements accurately becomes complex (Liu et al., 2017), and aspects, such as modality miss-matches are common (Zhang and Hua, 2015). Additionally, given the rapid developments in machine learning approaches, outdated methods may be held as benchmarks for longer than is scientifically meaningful.

### 4.3. Confidence and Trust

In AI data, the terms *confidence and trust* are applied to ensure reliability, i.e., having *confidence* in the data results in deeper *trust* (Arnold et al., 2019). In this context, *trust* is a qualitative term, and although *confidence* can fall into these interpretations relating to enhanced moral understanding (Blass, 2018), the term *confidence* typically refers to a quantifiable measure to base *trust* on (Zhang et al., 2001; Keren et al., 2018).

[±] Not providing an overall *confidence* for resulting predictions, can result in a substantial risk to the user (Ikuta et al., 2003), i.e., if a trained network has an inherent bias, a *confidence* measure improve the transparency of this. Furthermore, to increase *trust* in AI, developers are attempting to replicate human-like characteristics, e.g., how robots walk (Nikolova et al., 2018). Adequately reproducing such characteristics, requires substantial data sources from refined demographics. This concern falls primarily into *Machine Ethics*, with the need for binary gender identifications (Baird et al., 2017), and the societal effect of doing so challenged (Jørgensen et al., 2018).

### 4.4. Explainability and Interpretability

Often referred to as XAI (eXplainable AI) and arguably at the core of the ethical debate in the field of AI is *explanabilty* and *interpretability*. These terms are synonymous for the need to understand algorithms' decision making (Molnar, 2019; Tjoa and Guan, 2019). However, a distinction can be made, *interpretability* being methods for better understanding a machine learning architecture or data source (i.e., the *how*), and *explainability* being methods for understanding *why* particular decision were made.

[±] A surge in machine learning research, has come from international challenges (Schuller et al., 2013; Ringeval et al., 2019)—driving improvements in accuracy across multiple machine learning domains (Meer et al., 2000). However, this fast-paced environment often leaves less time for interpreting how particular features may have explicitly impacted a result, or for an explanation of a models decision-making process. Without this, the meaning of any result is less easy to substantiate (Vellido et al., 2012).

## 5. Discussion: Representation and Infrastructure

Having defined our four key consideration more concretely, we now discuss them more closely with representation (w.r.t., bias) and infrastructure of AI data in mind. Where meaningful, we highlight technical approaches which are implemented to reduce the aforementioned ethical concerns.

### 5.1. Auditing

There are many methods being developed to make collecting and annotating data in an automatic way possible, including *data mining* of web-based images (Zafar et al., 2019), and *active learning* (AL) for semi-automatic labeling (Wang et al., 2019c). For data tagging by autonomous agents, some have shown concerns that making agents responsible for this, may lead to incorrect tagging caused by an initial human error. A concern which becomes more problematic given the now large quantities of child viewers, who may be *suggested* inappropriate content (Papadamou et al., 2019). Further to this when annotating data, one ethical issue which can propagate *selection-bias* is poorly balanced manual vs. automatic annotations. In other words, if automatic annotation procedures learn false aspects early on, these may then be replicated (Rothwell et al., 2015). In an AL paradigm (Ayache and Quénot, 2008), an *oracle* (i.e., expert auditor) is kept in the loop, and where the AL model is uncertain at a particular level of *confidence*, the oracle must provide the label (Settles et al., 2008). In the case of specialist domains, such as bird sound classification, having such an expert is crucial, as variances in the audio signal can be quite slight (Qian et al., 2017).

Within a larger *decentralized* network, utilizing auditors allows for a democratic style of data management. Blockchain AI networks, for example, run in a peer-to-peer (P2P) fashion, meaning that no changes can be made to the system without the agreement of all others in the network. In a P2P network, there is an incentive for individual participation in the *auditing* process (e.g., an improved overall experience) (Dinh and Thai, 2018). However, the realization of *auditing* in AI does lead to some technical challenges in regards to public verification of sensitive data (Diakopoulos and Friedler, 2017), as well as making the AI only a partial reduction of human time-cost. Nevertheless, the need for *auditing* in AI has been highlighted consistently in the literature as a bias mitigating approach (Saleiro et al., 2018)

### 5.2. Benchmarking

It has been noted in many domains of research that *benchmarking* and therefore generalizing against a well-established organization, may result in the continued propagation of poor standards concerning historical biases (Denrell, 2005). Survey-based evaluations of the state-of-the-art modalities and baselines results are one resource to help mitigate this issue (Liu et al., 2011; Cummins et al., 2018). However, constant updates to benchmarks should be made, updating both techniques for acquisition and methods for setting baselines. Although there is no rule of thumb in this case, it is generally accepted in machine learning that *benchmarking* against resources that are no longer considered to be state-of-the-art will not bring valid results. Furthermore, in the realm of human-data, and specifically within the European Union, there is often a limited time that data can be stored (The-European-Commission, 2019). In this way, not only will benchmarked data sets become outdated in terms of techniques, but it is unethical to utilize such data, as reproducibility may not be possible.

Of note, a considerable contribution for ethics-based *benchmarking* is the aforementioned open-source IBM AI Explainability 360 Toolkit, in which one aspect is the Adversarial Robustness 360 Toolbox. This toolbox provides state-of-the-art paradigms for adversarial attacks (i.e., subtle alterations to data), and allows researchers to benchmark their approaches in a controlled environment to allow for more easy *interpretation* of possible network issues.

### 5.3. Confidence and Trust

Given the general fear that members of the public have for AI—mostly attributed to false depictions in movies and literature – improving *confidence and trust* in AI is now at the forefront for many corporations. To this end, researchers and corporations continually introduce state-of-the-art aids for tackling famous AI problems, such as the IBM AI Fairness 360 Toolkit. As well as this, to improve *trust* groups, such as “IBM Building Trust in AI”^7^, make this their specific focus. In this particular group, developing human-like aspects is given a priority, as research has shown that humans *trust* the general capability of more human-like representations over purely mechanical ones (Charalambous et al., 2016). However, the well-known uncanny valley (which refers to familiarity and likeability, concerning human-likeness) suggests that data-driven representations requiring *trust* should be very-near human-like (Mori et al., 2012), and action may result in biased binary representations, which may be problematic in terms of identity politics (Jørgensen et al., 2018).

Another effort in improving *trust* comes from blockchain. Blockchain is a specific *decentralized* approach known as a distributed digital ledger, in which transactions can only be altered with the specific agreement of subsequent (connected) blocks (Zheng et al., 2018). Blockchain is said to offer deeper *trust* for a user within a network, due to the specific need for collaboration (Mathews et al., 2017). This approach offers further accountability, as decisions, or alterations are agreed upon by those within the network. More specifically, *trust* is established through algorithms known as consensus algorithms (Lee, 2002).

As mentioned, one quantifiable measure to build on *trust* are *confidence measures*, sometimes referred to as *uncertainty measures* i.e., those applied in a semi-automated labeling paradigm. A *confidence* measure evaluates the accuracy of a model's predictions against a ground truth or set of weights and provides a metric of *confidence* in the resulting prediction (Jha et al., 2019). Herein, we follow this definition for *confidence* as a measure, i.e., how accurate is the current system prediction, as a means of understanding any risk (Duncan, 2015). This definition allows researchers to have a margin of error and can be a crucial aspect of the health domain to avoid false-positives (Bechar et al., 2017).

Given the “black-box” nature of deep learning, there have been numerous approaches to quantifying *confidence* (Kendall and Cipolla, 2016; Keren et al., 2018). One popular procedure for measuring *confidence* is the *Monte Carlo dropout*. In this approach, several iterations are made, each time “dropping” a portion of the network, and calculating *confidence* or uncertainty based on the variance of each prediction (Gal and Ghahramani, 2016).

As an additional note, *data-reliability* is a term often referred to in regards to both *confidence and trust*. Typically this is the process of statistically representing the significance of any findings from the database in a well-established scientific fashion, particularly considering the context of the domain it is targeted toward (Morgan and Waring, 2004). Statistical tests, such as the *p*-value, which is used across research domains, including machine learning, remains controversial. A *p*-value, states the strength (significance) of evidence provided and suffers from the “dancing *p*-value phenomena” Cumming (2013). This phenomenon essentially shows that in a more real-world setting the *p*-value can range (within the same experimental settings) from <0.001 to 0.5, i.e., from very significant to not significant all. Given this limitation, the researcher may present a biased experiment, in an endeavor to report a significant result. This limitation of the *p*-value, amongst other statistical tests, has gained criticism in recent years, due to their extensive misuse by the machine learning community (Vidgen and Yasseri, 2016).

### 5.4. Explainability and Interpretability

Researchers continue to work towards more accurately understanding the decisions made by deep networks (Huszár, 2015; Rai, 2020). Machine learning models must be interpretable and offer a clear use-case. At the core of this, data itself in such systems should also be explainable i.e., designed data acquisition, with plausible goals. Machine learning is a pattern recognition task, and due to this visualization of data is one way to help with detailing both *interpretability* and *explainability* of a system by (1) better understanding the feature space, and (2) better understanding possible choices. In regards to the bias in AI, visualization of data-points allows for a more easily determined observation of any class dominance. Clustering is a particular pre-processing step applied in *Big Data*-based deep learning (Samek et al., 2017). Popular algorithms which apply this type of visualization include *t*-distributed stochastic neighbor embedding (*t*-SNE) (Zeiler and Fergus, 2014) and Laplacian Eigenmaps (Schütt et al., 2019). More recently, there has been a surge in approaches for visualizing attention over data points (Guo et al., 2019). These approaches are particularly promising as they show visually the areas of activation which are learnt most consistently for each class by a network (Wang et al., 2019b), therefore highlighting areas of bias more easily, and improving communication methods to those outside the field.

To this end, *decentralization* with integrated blockchain is one approach which has been noted as improving *interpretability*, mainly as data is often-publicly accessible (Dinh and Thai, 2018). For example, where bias begins to form, the diversity of modalities and ease in identification means that individual blocks can be excluded entirely from a network to meet a more accurate representation (Dai et al., 2019).

## 6. Future Directions

Due in part to the ethics-based commitments by some of the larger AI companies, we see from this review that, there is momentum toward a more ethical AI future. However, **interdisciplinarity** in AI research is one aspect which requires more attention. To the best of the authors' knowledge, most public forums (particularly those based on a centralized infrastructure) come from a mono-domain viewpoint (e.g., engineering). Incorporating multiple disciplines in the discussion appears to be more prominent with those promoting *decentralized* AI.

Interdisciplinary will not only improve implementation of the four ethical consideration described herein, but has been shown to be a necessary step forward for the next AI phase of Artificial General Intelligence (AGI), proposed by the decentralized community (Goertzel and Pennachin, 2007). Interdisiplinarity is particularly of value as infrastructures developed in this way more easily tackle ethical concerns relating to; (i) integration, (ii) *selection-bias*, and (iii) *trust*.

Seamless **integration** of AI is necessary for its success and adoption by the general public. Aspects including cultural and environmental impact need to be considered, and various experts should provide knowledge on the target area. For example, the synthesized voice of bus announcements not representing the community to which it speaks may have a negative impact on those communities, and a closer analysis of the voice that best represents that community would be more ethically considerate. In this way, working alongside linguists and sociologists may aid development.

Similarly, from our literature overview, we observe that knowledge of **selection-bias** often requires contributions from experts with non-technical backgrounds, and an approach for facilitating discussion between fields of research would be a valuable next step. For example, within the machine learning community, techniques, such as *few-shot learning* are receiving more attention in recent years (Wang and Yao, 2019), however, perceptual-based biases pose difficulties for such approaches (Azad et al., 2020), and discussion from experts of the targeted domains may help understand the bias at an earlier stage. Despite this, communication between fields speaking different “languages” (i.e., anthropology and engineering), is a challenge in itself, which should be addressed by the community. Furthermore, due to historical stereotypes, AI continues to lack in **trust** by the general user. Users who without an understanding of the vocabulary of the field, may not be able to grasp the concept of such networks. Through a better collaboration with various academic researchers, communicating AI to the general public may also see an improvement, which in turn will help to build trust and improve wellbeing of the user during AI interaction.

## 7. Conclusion

The themes of data representation and infrastructure as they pertain to *selection-bias* and *decentralization* in AI algorithms have been discussed throughout this contribution. Within these discussion points, we have highlighted four key consideration; *auditing, benchmarking, confidence and trust*, and *explainability and interpretability* to be taken into account when handling AI data more ethically.

From our observation, we conclude that for all of the four considerations, issues which may stem from multimodal approaches should be treated cautiously. In other words, relating to *auditing*, there should be standards for each modality monitored, as this follows through into the ability for accurate *benchmarking*. In this same way, although the literature may argue this, *confidence and trust* come from diverse representations of human data, which in turn are more *explainable* to the general public due to its inherent human-like attributes.

With this in mind, we see that efforts are being made, for fully audited, benchmarkable, confident, trustworthy, explainable and interpretable machine learning approaches. However, standardization for the inclusion of all of these aspects is still needed. Furthermore, with the inclusion of multiple members who take equal responsibility, *decentralization* may enable the ethical aspects highlighted herein. We see that through social-media (which is in some sense a decentralized network for communication) group morality is developed. Opinions of a political nature, for example, are highlighted, and any prejudices or general wrongdoing is often shunned and which can have enormous impact on business (Radzik et al., 2020). In this way, a more transparent and open platform makes masking potential network biases a challenge.

## Author Contributions

AB: literature analysis, manuscript preparation, editing, and drafting manuscript. BS: drafting manuscript and manuscript editing. All authors revised, developed, read, and approved the final manuscript.

## Conflict of Interest

The authors declare that the research was conducted in the absence of any commercial or financial relationships that could be construed as a potential conflict of interest.

## Footnotes

^1^DeepMind : https://deepmind.com/applied/deepmind-ethics-society/. Partnership on AI: https://www.partnershiponai.org/board-of-directors/.

^2^Google: https://developers.google.com/machine-learning/fairness-overview/.

^3^IBM AI Explainability 360 Toolkit: https://www.research.ibm.com/artificial-intelligence/trusted-ai/.

^4^Effect.AI: https://effect.ai/., SingularityNET https://singularitynet.io/.

^5^Decentralized AI Alliance: https://daia.foundation/.

^6^The Algorithm Justice League: https://www.ajlunited.org/, and the AI NOW institute https://ainowinstitute.org/.

^7^IBM—Building Trust in AI: https://www.ibm.com/watson/advantage-reports/future-of-artificial-intelligence/building-trust-in-ai.html.
